# Supplementary material for: Eliminating chronic myeloid leukemia stem cells by IRAK1/4 inhibitors
Source: Nat Commun. 2022 Jan 12;13:271. doi: 10.1038/s41467-021-27928-8 (PMC8755781; doi:10.1038/s41467-021-27928-8)
Supplement: Supplementary file 1 — Supplementary Information [file 41467_2021_27928_MOESM1_ESM.pdf]

Supplementary Information

Eliminating chronic myeloid leukemia stem cells by IRAK1/4 inhibitors

Yosuke Tanaka[1,†], Reina Takeda[1], Tsuyoshi Fukushima[1], Keiko Mikami[1], Shun Tsuchiya[2], Moe Tamura[1], Keito Adachi[1], Terumasa Umemoto[5], Shuhei Asada[1], Naoki Watanabe[2], Soji Morishita[3], Misa Imai[3], Masayoshi Nagata[4], Marito Araki[3], Hitoshi Takizawa[5], Tomofusa Fukuyama[1], Chrystelle Lamagna[6], Esteban S Masuda[6], Ryoji Ito[7], Susumu Goyama[8], Norio Komatsu[2], Tomoiku Takaku[2], Toshio Kitamura[1,†]

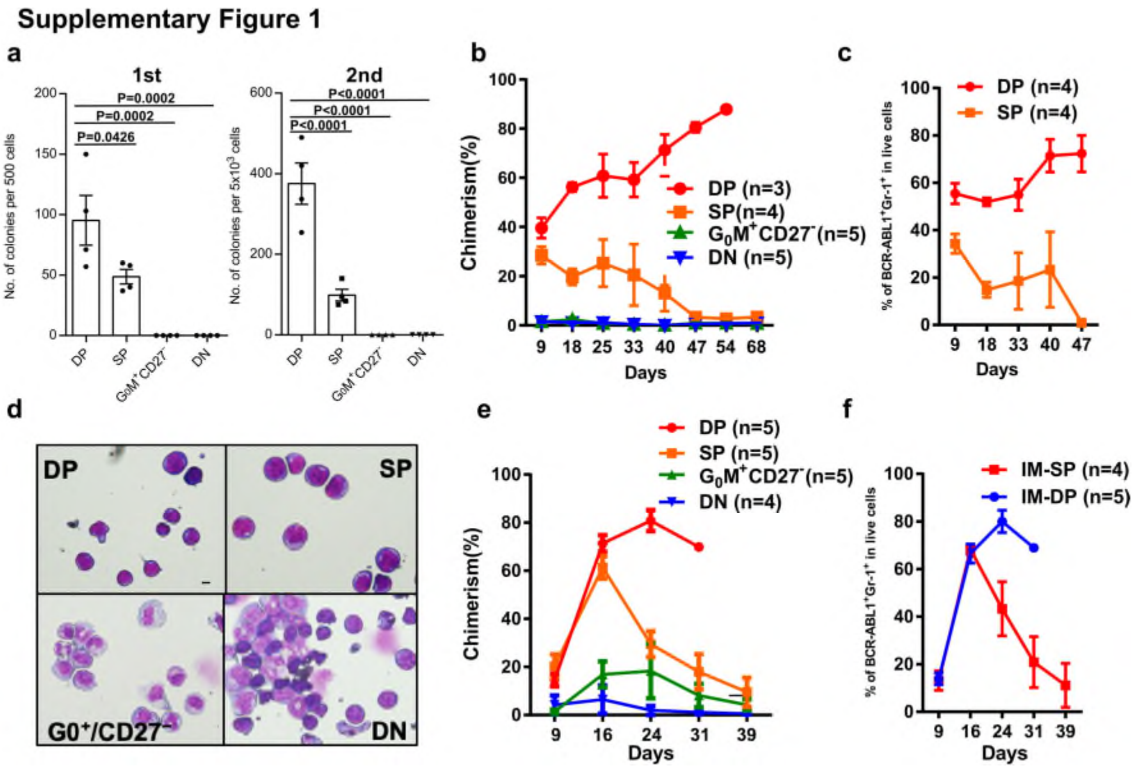

Supplementary Figure 1. Identification of quiescent CML LSCs by G_0M, related to Figure 1

a Colony-forming assays of the indicated populations from vehicle-treated CML mice. 500 cells were plated in the 1st round (left), and $5x10^3$ cells were plated in the 2nd round (right). n=4 each. **b** Kinetic analysis of the chimerism of GFP^+ cells in the peripheral blood (PB) of recipient mice transplanted with the indicated populations from vehicle-treated CML mice. **c** Kinetic analysis of the chimerism of $GFP^+Gr\text{-}1^+$cells in the PB of recipient mice transplanted with the indicated populations from vehicle-treated CML mice. **d** Giemsa marrow cytospins of the indicated populations from untreated CML mice. Scale bar, 5 µm. Representative pictures were shown from n=2 independent experiments. **e** Kinetic analysis of chimerism of GFP^+ cells in the peripheral blood of recipient mice transplanted with the indicated populations from imatinib-treated CML mice. DP (n=5), SP (n=5), $G_0M^+CD27^-$ (n=5), and DN (n=4). **f** Kinetic analysis of the chimerism of $GFP^+Gr\text{-}1^+$cells in the peripheral blood of recipient mice transplanted with the indicated populations from imatinib-treated CML mice. Data are shown as the mean ± SEM. P values are calculated using one-way ANOVA with Tukey's correction for multiple comparisons (**a**)

Supplementary Figure 2

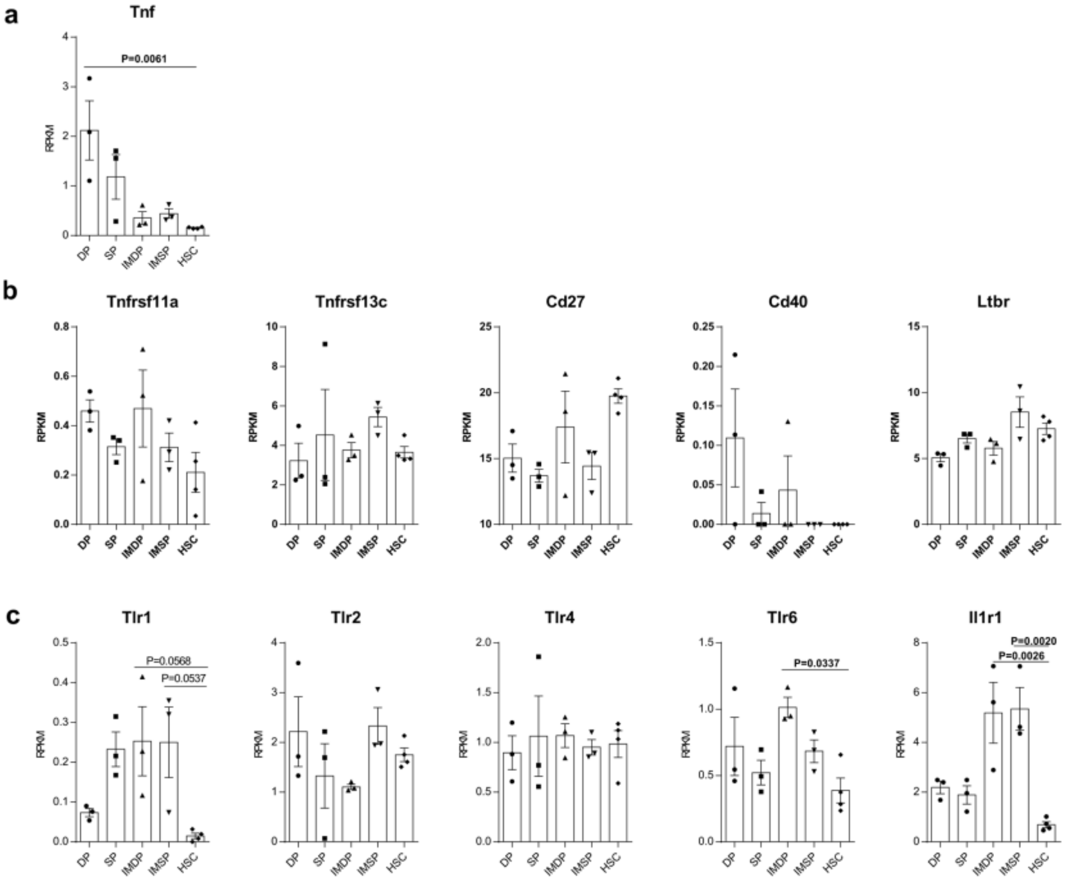

Supplementary Figure 2. TLRs and IL1R1 are highly expressed in imatinib-insensitive CML LSCs, related to Figure 2

The RPKMs of *Tnf* (**a**), receptors on noncanonical NF-κB (**b**) and Toll-like receptors (*Tlr1*, *Tlr2*, *Tlr4* and *Tlr6*) and *Il1r1* (**c**) among the indicated CML populations (n=3 each) and normal HSCs (n=4). Data are shown as the mean ± SEM. P values are calculated using one-way ANOVA with Tukey's correction for multiple comparisons (**a-c**).

Supplementary Figure 3

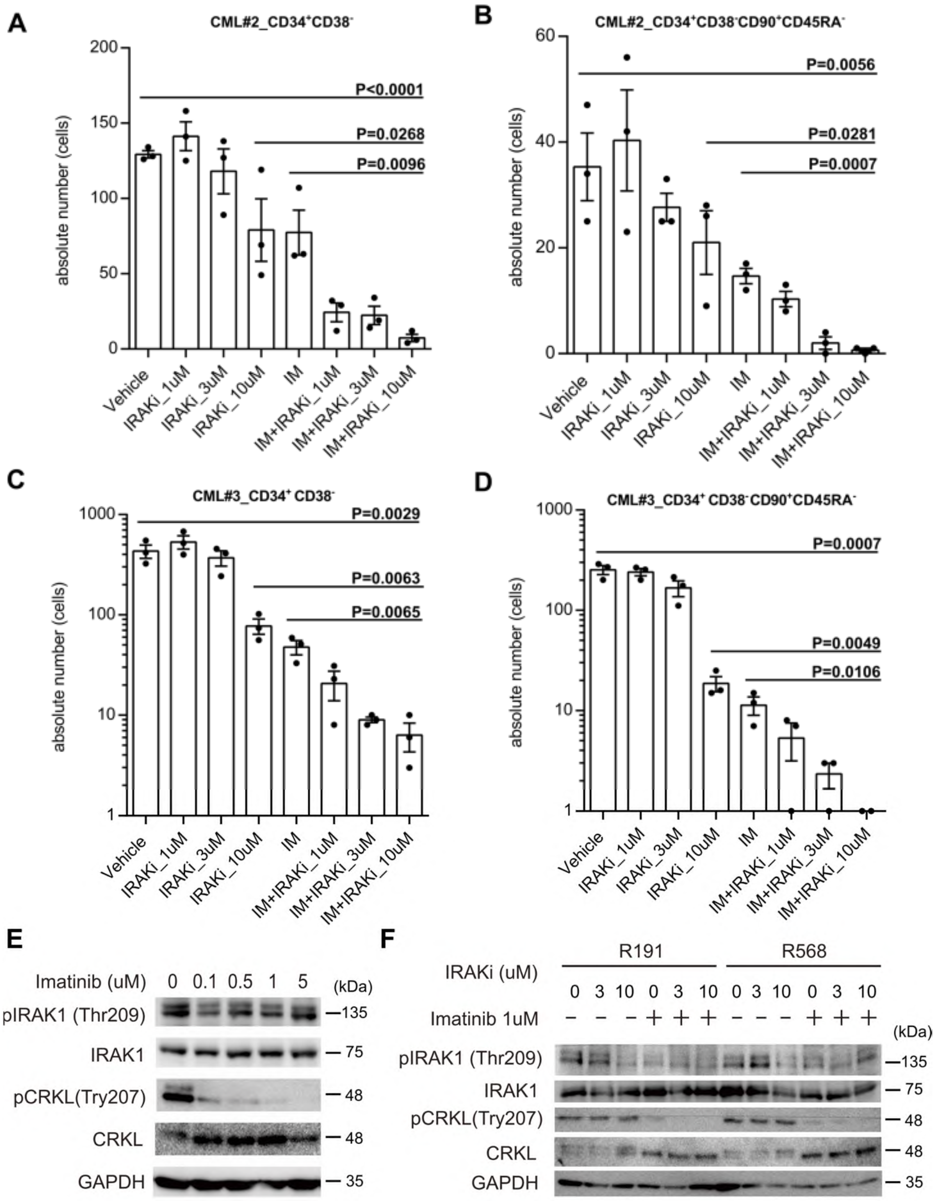

Supplementary Figure 3. The combination of IRAK1/4 inhibitor and imatinib is detrimental to CML-CP CML LSCs, related to Figure 4

a-d MACS-purified CD34$^+$ stem/progenitor cells (1×10^2) from the BM of newly diagnosed CML-CP patients (n=2) were cultured in liquid culture in the presence of imatinib (IM: 0.5 μM), IRAK1/4 inhibitor (IRAKi (R568): 1, 3 or 10 μM), or the combination of for one week (n=3 each). The absolute number of CD34$^+$CD38$^-$ cells (**a** and **c**) and CD34$^+$CD38$^-$CD90$^+$CD45RA$^-$ cells (**b** and **d**). **e** and **f** Protein lysates from K562 cells treated with increasing concentrations of imatinib (**e**) and increasing concentrations of IRAKi (R568 and R191) with and without 1μM imatinib (**f**) for 2 hours

were evaluated by immunoblotting for IRAK1, pIRAK1T209, CRKL and pCRKLT207. Representative data are shown from n=3 independent experiments. Data are shown as the mean ± SEM. P values were calculated using two-tailed Student's *t*-test (**a-d**).

Supplementary Figure 4

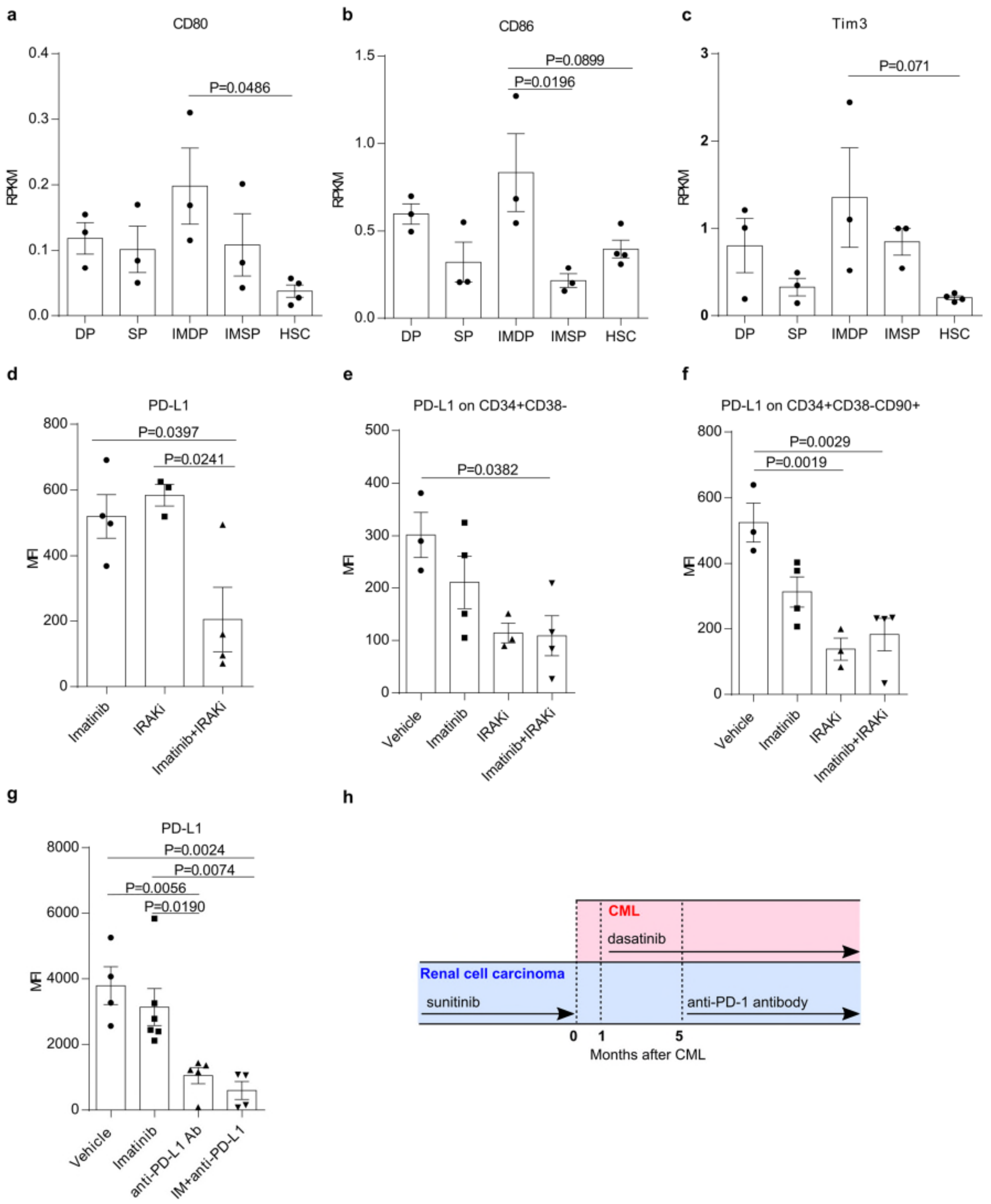

Supplementary Figure 4. Immune checkpoint molecules on CML LSCs, related to Figure 5

a-c The RPKMs of *Cd80*, *Cd86* and *Tim3* in the indicated CML populations (n=3 each) and normal HSCs (n=4). **d** The mean fluorescence intensity (MFI) of mouse PD-L1 on DP cells from CML mice treated with imatinib (n=4), IRAK1/4 inhibitor (n=3) or the combination (n=4). **e and f** The MFI of human PD-L1 on CD34^{+}CD38^{-} cells (**e**) and

$CD34^{+}CD38^{-}CD90^{+}$ cells (**f**) in the BM of CML-CP $CD34^{+}$ xenografts treated with vehicle (n=3), imatinib (n=4), IRAK1/4 inhibitor (IRAKi (R930259 Diet): 100 ppm) (n=3) or the combination (n=4). **g** The MFI of mouse PD-L1 on DP cells from CML mice treated with vehicle (n=4), imatinib (n=6), anti-PD-L1 antibody (n=5) or the combination (n=4). **h** A medical history of the double cancer patient. Data are shown as the mean ± SEM. P values are calculated using one-way ANOVA with Tukey's correction for multiple comparisons (**a-f**).

Supplementary Table 1: Characteristics of CML patient samples used in this study. Related to Methods.

CML patient No.	Sex	Age (range)	Samples used for experiments in
1	Female	20-29	Figures 4A-E
2	Male	50-59	Supplementary Figure 4A and 4B
3	Female	40-49	Supplementary Figure 4A and 4B
4	Male	60-69	Figures 4F-H

Supplementary Table 2: Characteristics of CML patients who received Dasatinib

CML patient No.	Age (range)	Treatment	Sokal Score
1	60-69	Dasatinib(100mg Once Daily)	0.76
2	40-49	Dasatinib(100mg Once Daily)	0.54
3	70-79	Dasatinib(100mg Once Daily)	0.81
4	40-49	Dasatinib(100mg Once Daily)	0.75
5	20-29	Dasatinib(100mg Once Daily)	0.45
6	80-89	Dasatinib(100mg Once Daily)	0.99
7	30-39	Dasatinib(100mg Once Daily)	0.52
8	50-59	Dasatinib(100mg Once Daily)	0.65
9	50-59	Dasatinib(100mg Once Daily)	0.59
10	70-79	Dasatinib(100mg Once Daily)	1.42
11	70-79	Dasatinib(100mg Once Daily)	1.11
12	40-49	Dasatinib(100mg Once Daily)	0.54
13	40-49	Dasatinib(100mg Once Daily)	0.64
14	30-39	Dasatinib(100mg Once Daily)	0.53
15	60-69	Dasatinib(100mg Once Daily)+Nivolumab	1.02
