# Supplementary material for: Eliminating chronic myeloid leukemia stem cells by IRAK1/4 inhibitors
Source: Nat Commun. 2022 Jan 12;13:271. doi: 10.1038/s41467-021-27928-8 (PMC8755781; doi:10.1038/s41467-021-27928-8)
Supplement: Supplementary file 3 — Reporting summary [file 41467_2021_27928_MOESM3_ESM.pdf]

nature research

Corresponding author(s): Yosuke Tanaka

Last updated by author(s): Dec 6, 2021

Reporting Summary

Nature Research wishes to improve the reproducibility of the work that we publish. This form provides structure for consistency and transparency in reporting. For further information on Nature Research policies, see our Editorial Policies and the Editorial Policy Checklist.

Statistics

For all statistical analyses, confirm that the following items are present in the figure legend, table legend, main text, or Methods section.

n/a	Confirmed	
☐	☒	The exact sample size (*n*) for each experimental group/condition, given as a discrete number and unit of measurement
☐	☒	A statement on whether measurements were taken from distinct samples or whether the same sample was measured repeatedly
☐	☒	The statistical test(s) used AND whether they are one- or two-sided<br>*Only common tests should be described solely by name; describe more complex techniques in the Methods section.*
☒	☐	A description of all covariates tested
☐	☒	A description of any assumptions or corrections, such as tests of normality and adjustment for multiple comparisons
☐	☒	A full description of the statistical parameters including central tendency (e.g. means) or other basic estimates (e.g. regression coefficient) AND variation (e.g. standard deviation) or associated estimates of uncertainty (e.g. confidence intervals)
☐	☒	For null hypothesis testing, the test statistic (e.g. F, t, r) with confidence intervals, effect sizes, degrees of freedom and P value noted<br>*Give P values as exact values whenever suitable.*
☒	☐	For Bayesian analysis, information on the choice of priors and Markov chain Monte Carlo settings
☒	☐	For hierarchical and complex designs, identification of the appropriate level for tests and full reporting of outcomes
☒	☐	Estimates of effect sizes (e.g. Cohen's d, Pearson's r), indicating how they were calculated

Our web collection on statistics for biologists contains articles on many of the points above.

Software and code

Policy information about availability of computer code

Data collection	Flow cytometry: BD FACSuite (1.0.5.3841) or BD FACSDiva (8.0.2) software<br>Immunofluorescence: EVOS FL Auto 2 Imaging System
Data analysis	Flow cytometry: FlowJo (10.4.2)<br>Immunofluorescence: Celleste (4.1)<br>RNA-seq: cutadapt (Galaxy Version 1.16.6), fastx-toolkit (0.0.13), HISAT2(Galaxy Version 2.1.0+galaxy7), featureCounts(Galaxy Version 2.0.1), edgeR (Galaxy Version 3.24.1+galaxy1), GSEA software (4.0.2), Enricher<br>Statistics: GraphPad Prism (6.0.0)

For manuscripts utilizing custom algorithms or software that are central to the research but not yet described in published literature, software must be made available to editors and reviewers. We strongly encourage code deposition in a community repository (e.g. GitHub). See the Nature Research guidelines for submitting code & software for further information.

Data

Policy information about availability of data

All manuscripts must include a data availability statement. This statement should provide the following information, where applicable:

- Accession codes, unique identifiers, or web links for publicly available datasets
- A list of figures that have associated raw data
- A description of any restrictions on data availability

Source data are provided with this paper. RNA-seq data are available in sequence read archive (SRA) database (accession ID:GSE175323). The datasets generated or analyzed during the current study are available from the corresponding author (ytims@ims.u-tokyo.ac.jp) on reasonable request.

Field-specific reporting

Please select the one below that is the best fit for your research. If you are not sure, read the appropriate sections before making your selection.

☒ Life sciences ☐ Behavioural & social sciences ☐ Ecological, evolutionary & environmental sciences

For a reference copy of the document with all sections, see nature.com/documents/nr-reporting-summary-flat.pdf

Life sciences study design

All studies must disclose on these points even when the disclosure is negative.

Sample size	Sample sizes were indicated in the legend of each Figure and Supplementary Figure. No statistical methods were used to predetermine sample sizes. We determined sample sizes as follows; for cell culture experiments, we performed at least triplicate experiments and for animal experiments, at least n=6 for each treatment group, to meet the minimal requirements for statistical analysis.
Data exclusions	No data were excluded from the analyses.
Replication	All experiments were replicated at least twice. All attempts to replication were successful.
Randomization	All animals, all patient cells and K562 cells were maintained in the same environment and were randomly assigned to the experimental groups.
Blinding	For data automatically collected by instruments, such as flow cytometry and RNA-seq analysis, researchers were not blinded as observer bias is expected not to affect the results. For data manually collected by researchers, such as counting nuclear p65, researchers were blinded during data analyses.

Reporting for specific materials, systems and methods

We require information from authors about some types of materials, experimental systems and methods used in many studies. Here, indicate whether each material, system or method listed is relevant to your study. If you are not sure if a list item applies to your research, read the appropriate section before selecting a response.

Materials & experimental systems

n/a	Involved in the study	
☐	☒	Antibodies
☐	☒	Eukaryotic cell lines
☒	☐	Palaeontology and archaeology
☐	☒	Animals and other organisms
☐	☒	Human research participants
☒	☐	Clinical data
☒	☐	Dual use research of concern

Methods

n/a	Involved in the study	
☒	☐	ChIP-seq
☐	☒	Flow cytometry
☒	☐	MRI-based neuroimaging

Antibodies

Antibodies used	(For flow cytometry analysis)<br><br>CD5 (BioLegend, 100604)<br>B220 (BioLegend, 103204)<br>CD11b (BioLegend, 101204)<br>Gr-1 (BioLegend, 108404)<br>Ter119 (BioLegend, 116204)

c-kit-PE-Cy7 (BioLegend, 105814)
Streptavidin-Brilliant Violet 605 (BioLegend, 405229)
Sca1-Brilliant Violet 785 (BioLegend, 108139)
Annexin V-APC (BioLegend, 640920)
CD274 (BioLegend, 124307)
CD27 (BioLegend, 124223)
CD34 (BioLegend, 343613)
CD38 (BioLegend, 356607)
CD90 (BioLegend, 328113)
CD45RA (BioLegend, 304105)
CD274 (BioLegend, 329705)
CD45 (BioLegend, 368507 and 368521)
Lineage Cocktail (BioLegend, 348807)

(For Immunofluorescence experiment)
NF-κB p65(Cell Signaling Technology, 8242)
Alexa Fluor 647 secondary antibody (Invitrogen)

(For in vivo blocking experiment)
PD-L1 (BioXcell, BE0101)
PD-1 (BioXcell, BP0273),
CTLA-4 (BioXcell, BE0164)

Validation

All antibodies are commercially available and those specificities were tested by manufactures. All vendors and catalog numbers of antibodies are listed above and detailed information is available on the websites. For flow cytometry analyses, specificity was evaluated using the proper control including isotype control antibody. The validation of each primary antibodies for the reactive species and applications are described below.

CD5 (BioLegend, 100604) - Reactive species: Mouse, Application: Flow cytometry
B220 (BioLegend, 103204) - Reactive species: Mouse, Human (Cross-Reactivity: Cat (Feline)), Application: Flow cytometry
CD11b (BioLegend, 101204) - Reactive species: Mouse, Human (Cross-Reactivity: Chimpanzee, Baboon, Cynomolgus, Rhesus, Rabbit (Lapine)), Application: Flow cytometry
Gr-1 (BioLegend, 108404) - Reactive species: Mouse, Application: Flow cytometry
Ter119 (BioLegend, 116204) - Reactive species: Mouse, Application: Flow cytometry
c-kit-PE-Cy7 (BioLegend, 105814) - Reactive species: Mouse, Application: Flow cytometry
Sca1-Brilliant Violet 785 (BioLegend, 108139) - Reactive species: Mouse, Application: Flow cytometry
Streptavidin-Brilliant Violet 605 (BioLegend, 405229) - Human, Mouse, Rat, All Species, Application: Flow cytometry
Annexin V-APC (BioLegend, 640920) - Reactive species: All mammalian species, Application: Flow cytometry
CD274 (BioLegend, 124307) - Reactive species: Mouse, Application: Flow cytometry
CD27 (BioLegend, 124223) - Reactive species: Mouse, Rat, Human, Application: Flow cytometry
CD34 (BioLegend, 343613) - Reactive species: Human (Cross-Reactivity: Cynomolgus, Rhesus), Application: Flow cytometry
CD38 (BioLegend, 356607) - Reactive species: Human, Application: Flow cytometry
CD90 (BioLegend, 328113) - Reactive species: Human, African Green, Baboon, Cynomolgus, Pigtailed Macaque, Rhesus, Swine (Pig, Porcine), Application: Flow cytometry
CD45RA (BioLegend, 304105) - Reactive species: Human (Cross-Reactivity: Chimpanzee), Application: Flow cytometry
CD274 (BioLegend, 329705) - Reactive species: Human, African Green, Baboon, Cynomolgus, Rhesus, Application: Flow cytometry
CD45 (BioLegend, 368507 and 368521) - Reactive species: Human, Application: Flow cytometry
Lineage Cocktail (BioLegend, 348807) - Reactive species: Human, Application: Flow cytometry
NF-κB p65(Cell Signaling Technology, 8242) - Reactive species: Human Mouse, Rat, Hamster, Monkey, Dog, Application: Immunofluorescence
PD-L1 (BioXcell, BE0101) - Reactive species: Mouse, Application: in vivo blocking
PD-1 (BioXcell, BP0273) - Reactive species: Mouse, Application: in vivo blocking
CTLA-4 (BioXcell, BE0164) - Reactive species: Mouse, Application: in vivo blocking

Eukaryotic cell lines

Policy information about cell lines

Cell line source(s)

State the source of each cell line used.

Authentication

Describe the authentication procedures for each cell line used OR declare that none of the cell lines used were authenticated.

Mycoplasma contamination	*Confirm that all cell lines tested negative for mycoplasma contamination OR describe the results of the testing for mycoplasma contamination OR declare that the cell lines were not tested for mycoplasma contamination.*
Commonly misidentified lines (See ICLAC register)	*Name any commonly misidentified cell lines used in the study and provide a rationale for their use.*

Animals and other organisms

Policy information about studies involving animals; ARRIVE guidelines recommended for reporting animal research

Laboratory animals	Wild-type C57BL/6J mice were bred in-house. Conditional G0 marker KI mice have been generated in our laboratory, crossed to Vav1-Cre transgenic mice and maintained as Ly5.1 genetic background. Rag2 knockout mice (Ly5.1 genetic background) were purchased from Sankyo Labo Service Corporation,INC. Six to 12-week-old female mice were used for all experiments.
Wild animals	No wild animals were used in this study.
Field-collected samples	No field-collected samples were used in this study.
Ethics oversight	The experiments were approved by the Committee on the Ethics of Animal Experiments and all these mice were maintained according to the guidelines of the Institute of Laboratory Animal Science (PA13-19 and PA16-31).

Note that full information on the approval of the study protocol must also be provided in the manuscript.

Human research participants

Policy information about studies involving human research participants

Population characteristics	Patient information was described in Supplemental Table 1 &2.
Recruitment	All CML patients in this study were from Juntendo University hospital and under standard treatments. They were not in any clinical trials.
Ethics oversight	All experiments were performed according to the Declaration of Helsinki and were approved by the Ethics Committee of Juntendo University School of Medicine (IRB#2019113) and the Research Ethics Review Committee of the Institute of Medical Science of the University of Tokyo (2019-8-0702).

Note that full information on the approval of the study protocol must also be provided in the manuscript.

Flow Cytometry

Plots

Confirm that:

- [x] The axis labels state the marker and fluorochrome used (e.g. CD4-FITC).
- [x] The axis scales are clearly visible. Include numbers along axes only for bottom left plot of group (a 'group' is an analysis of identical markers).
- [x] All plots are contour plots with outliers or pseudocolor plots.
- [x] A numerical value for number of cells or percentage (with statistics) is provided.

Methodology

Sample preparation	Bone marrow cells were obtained by flushing long bones (femurs and tibias) in phosphate-buffered saline (PBS) containing 2% heat-inactivated FBS (FACS buffer). Spleen cells were obtained by mechanical digestion n phosphate-buffered saline (PBS) containing 2% heat-inactivated FBS (FACS buffer). Cell suspensions were lysed with erythrocyte lysis buffer (150 mM NH4Cl, 10 mM KHCO3, 100 µM EDTA-Na2), filtered through a 40 µm filter. Cells were then stained with fluorochrome-conjugated antibodies. F
Instrument	Flow cytometry experiments were performed using FACSVerse or FACSAria.
Software	Data were collected with BD FACSDiva software and were analyzed with FlowJo software.
Cell population abundance	Cell populations were sorted to >95% purity post sort in pilot experiments, as determined by flow cytometry.
Gating strategy	Gating strategies are shown in Supplementary Figure 1. In brief, cells were gated for size exclusion (FSC-A/SSC-A) followed by doublet exclusion (FSC-H/FSC-W and SSC-H/SSC-W). The following gating were performed as indicated in Supplementary Figure 1. Boundaries between negative and positive were determined by single stained control.

- [x] Tick this box to confirm that a figure exemplifying the gating strategy is provided in the Supplementary Information.
